# Enteroenteric Fistula: A Rare Sequela of Unwitnessed Magnet Ingestion in a Child

**DOI:** 10.7759/cureus.81950

**Published:** 2025-04-09

**Authors:** Dalia D Abdulrahman, Avinash Hiremath, Ghadir Jaber, Mamoun M Al Marzouqi, Diary Mohammed

**Affiliations:** 1 Pediatric Surgery, Dubai Medical University, Dubai, ARE; 2 Pediatric Surgery, Al Jalila Children's Specialty Hospital, Dubai, ARE

**Keywords:** diagnostic laparoscopy, enteroenteric fistula, foreign body ingestion, intestinal obstruction, magnet beads

## Abstract

Enteroenteric fistula is an abnormal connection between two loops of bowel, commonly caused by chronic inflammatory disease, malignancy, history of a surgical procedure, radiation, or foreign body ingestion.

A previously healthy three-year-old female presented to the Emergency Department in Al Jalila Children's Specialty Hospital, Dubai, UAE, with a one-day history of non-bilious vomiting and colicky abdominal pain. She had no history of fever, loose stools, or bloody stools. On examination, her abdomen was soft, mildly distended, and tender all over, with no palpable masses. She was admitted for one day, improved, and was discharged in good general condition. However, 72 hours later, she presented with the same complaint, and the X-ray of the abdomen showed signs of intestinal obstruction, which were confirmed by an abdominal CT scan. A laparoscopy was performed, showing a band extending from the mid-ileum to the cecum and an enteroenteric fistula between the terminal ileum and cecum. The band was divided, and a laparoscopic-assisted division of the fistula was performed and sutured. She was discharged four days postoperatively in good general condition. On further questioning, the parents mentioned having a missing magnet at home.

This case highlights the serious risks of unnoticed magnet ingestion in children, emphasizing the importance of vigilance, prompt evaluation, and intervention to prevent complications, particularly in households with accessible small magnets. To our knowledge, this is the first reported case of an unwitnessed magnet ingestion, which was also not detected by imaging, causing an enteroenteric fistula and subsequently passing unnoticed.

## Introduction

Enteroenteric fistula is an abnormal connection between two loops of bowel, either small or large bowel. In most cases, there is an underlying disease, such as a chronic inflammatory disease or malignancy, or there is a history of a surgical procedure or radiation. In some cases, other causes of fistula formation are foreign bodies, which can be found through investigation using imaging studies [1]. To our knowledge, this is the first reported case of unwitnessed magnet ingestion, which was also not detected by imaging, causing an enteroenteric fistula and passing unnoticed.

## Case presentation

A previously healthy three-year-old female presented to the Emergency Department at Al Jalila Children’s Specialty Hospital, Dubai, UAE, with a one-day history of non-bilious vomiting and colicky abdominal pain. She had no history of fever, or loose or bloody stools.

**Figure 1:**
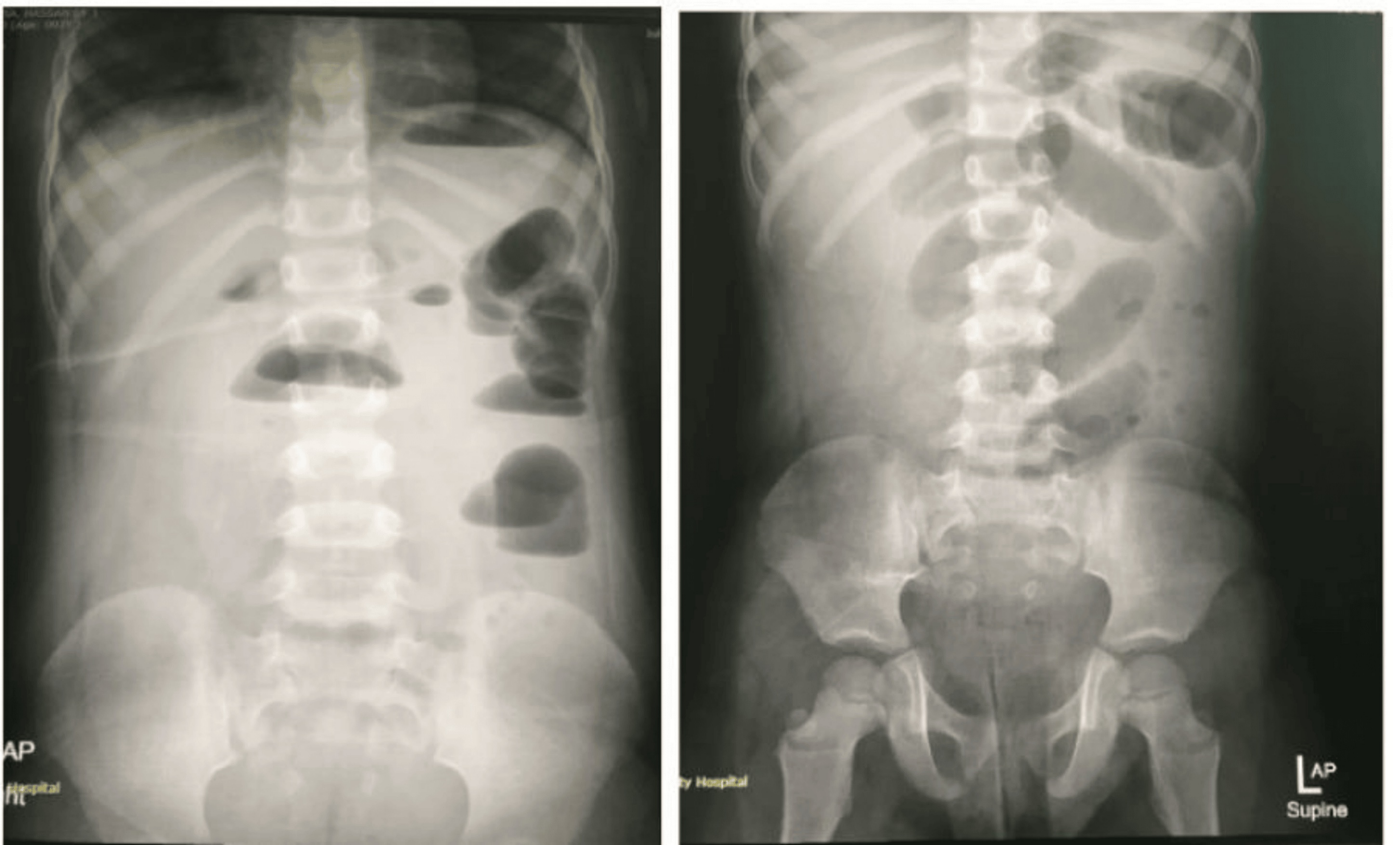
Day 1 abdominal X-rays (erect and supine) show dilated small bowels with multiple air fluid levels.

**Figure 2:**
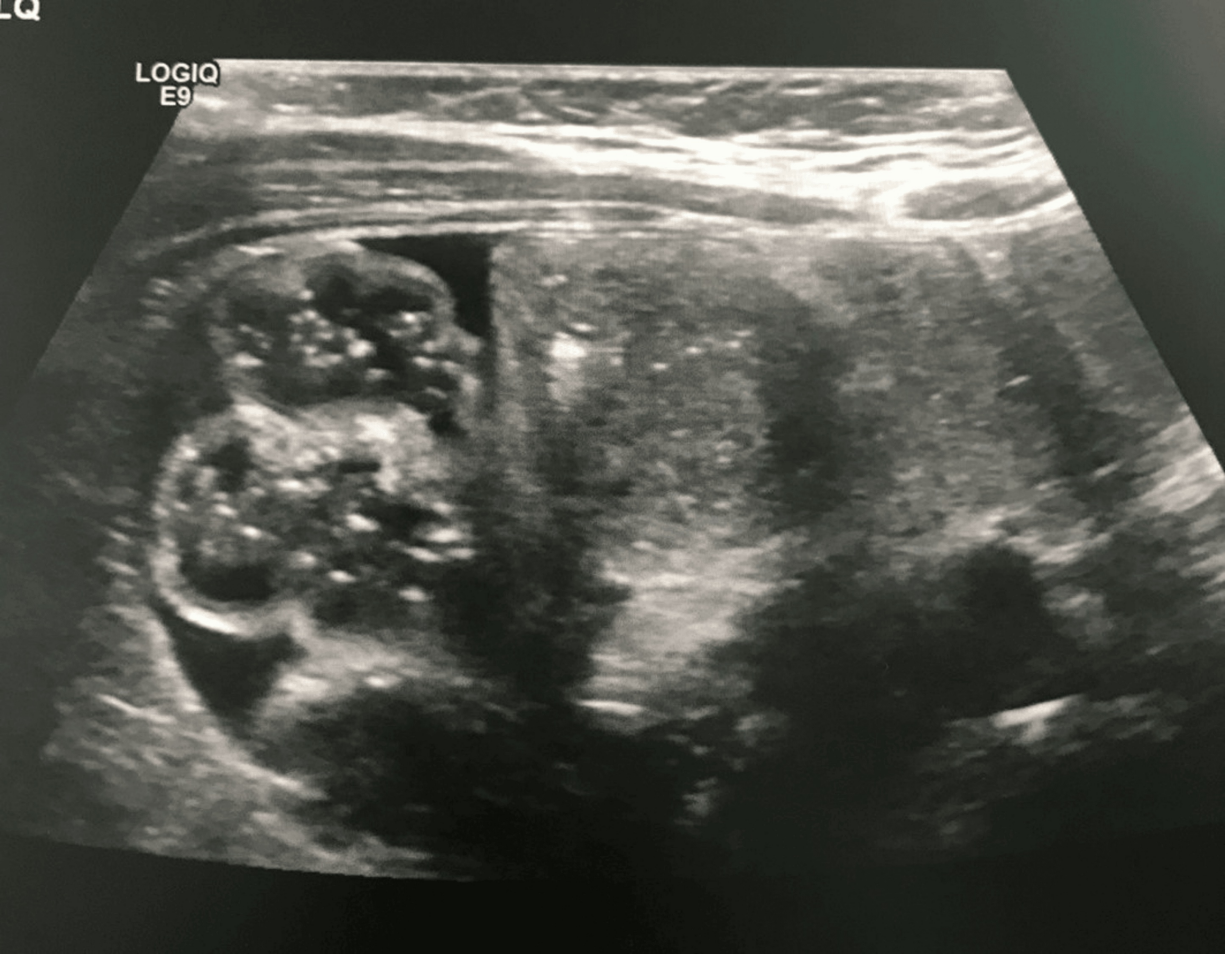
Day 1 abdominal ultrasound shows dilated bowel loops, and no masses are seen.

On examination, her abdomen was soft, mildly distended, and tender all over, with no palpable masses. She was admitted to the hospital under observation and was kept nil by mouth. Blood investigations were normal, and the plain abdominal X-ray showed multiple air-fluid levels suggestive of intestinal obstruction (Figure 1). An ultrasound of the abdomen showed dilated small bowel loops, with no masses or intussusception detected (Figure 2).

**Figure 3:**
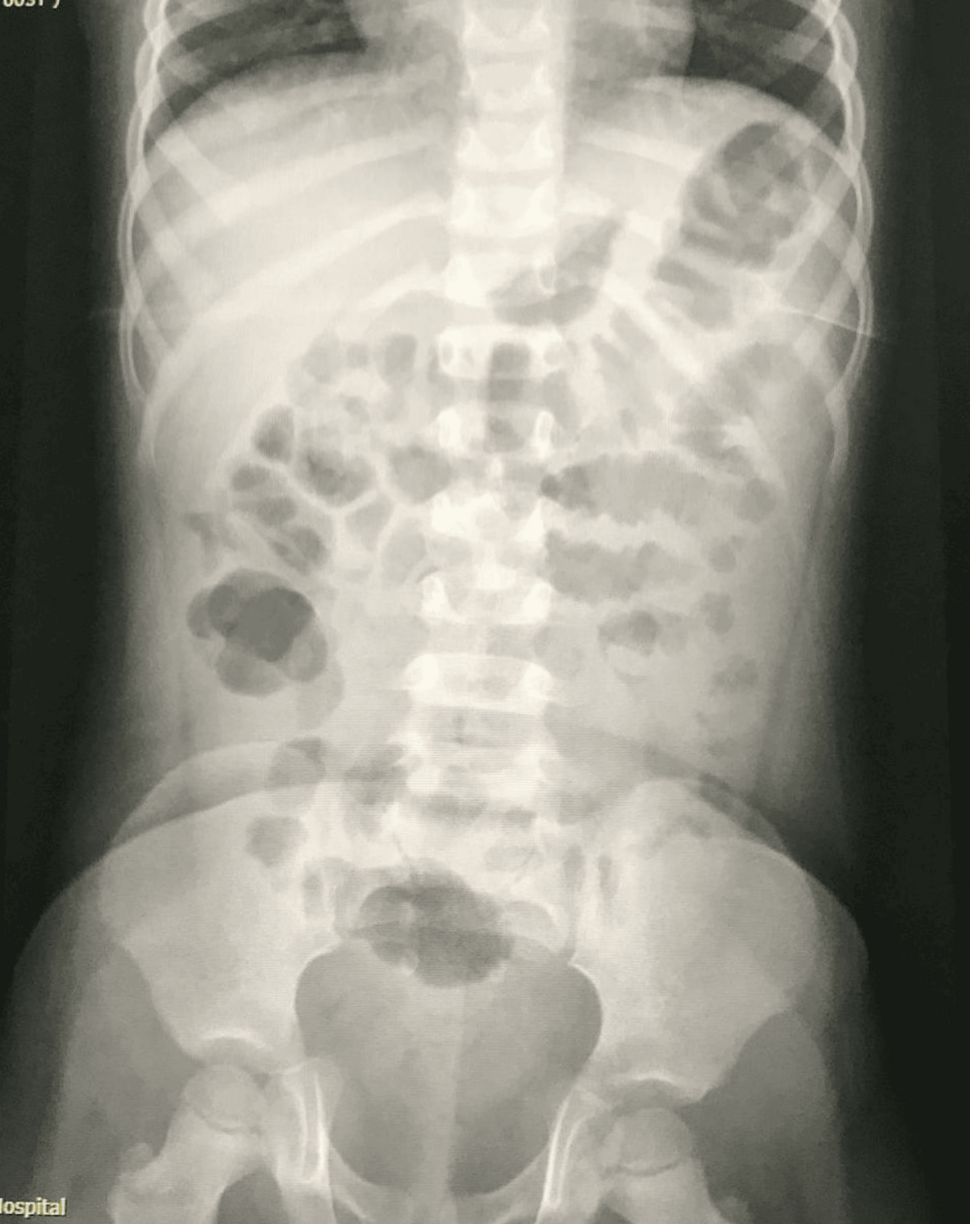
Day 2 normal abdominal X-ray (supine) with normal gas distribution.

The next day, she improved, with no abdominal pain or vomiting, and passed normal stools. She was started on oral fluids, then a soft diet, and tolerated it well, so she was discharged in good general condition (Figure 3).

**Figure 4:**
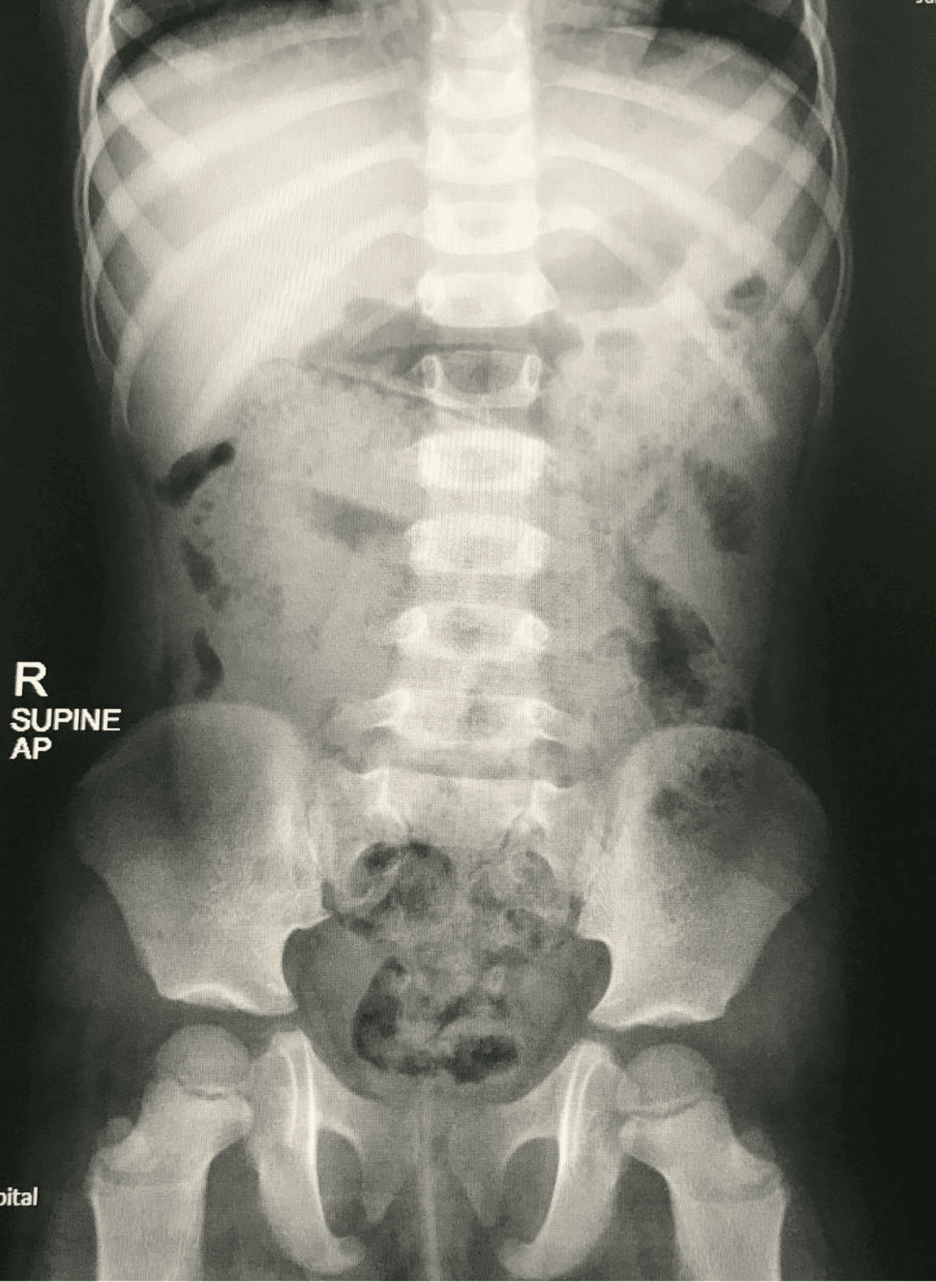
On readmission, 72 hours later, abdominal X-ray (supine) shows fecalization of the small bowel.

**Figure 5:**
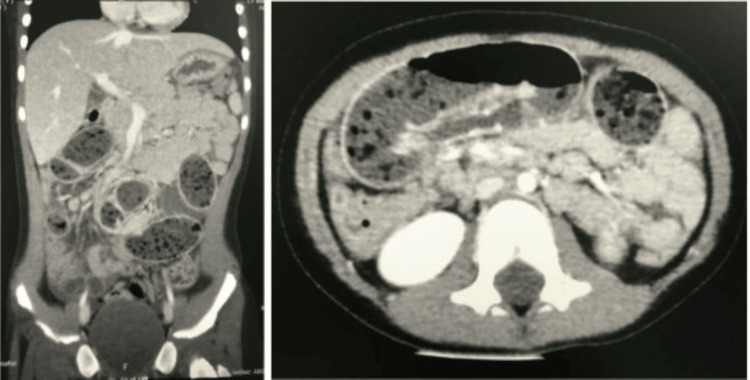
On readmission, CT abdomen (coronal and axial view) shows dilated proximal small bowel and collapsed distal bowel indicating intestinal obstruction.

**Figure 6:**
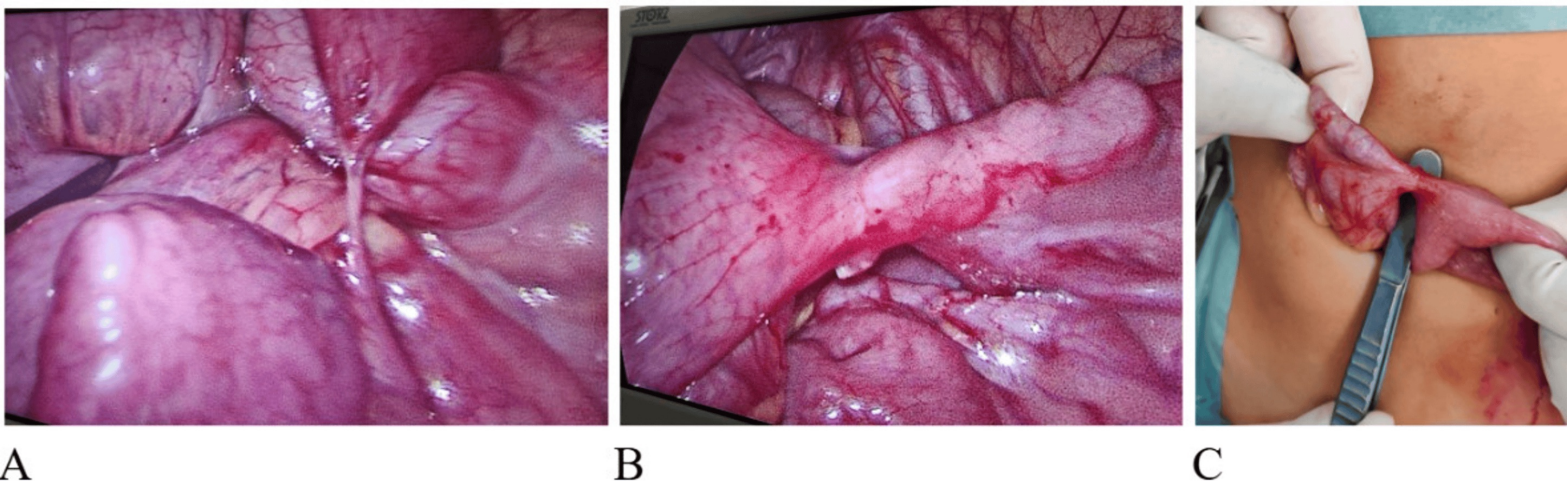
On readmission, intraoperatively, laparoscopy shows a band (A) extending from the mid-ileum to the cecum, causing obstruction, with an enteroenteric fistula (B and C) between the terminal ileum and the cecum.

After 72 hours, she was readmitted with the same complaint. An X-ray of the abdomen showed signs of intestinal obstruction (Figure 4), which was confirmed by an abdominal CT scan (Figure 5). The patient was booked for laparoscopic surgery, and it was found that there were dilated small bowel loops, with a band extending from the mid-ileum to the cecum and an enteroenteric fistula between the terminal ileum and cecum. The band was divided, and laparoscopic-assisted division of the fistula was performed and sutured (Figure 6).

**Figure 7:**
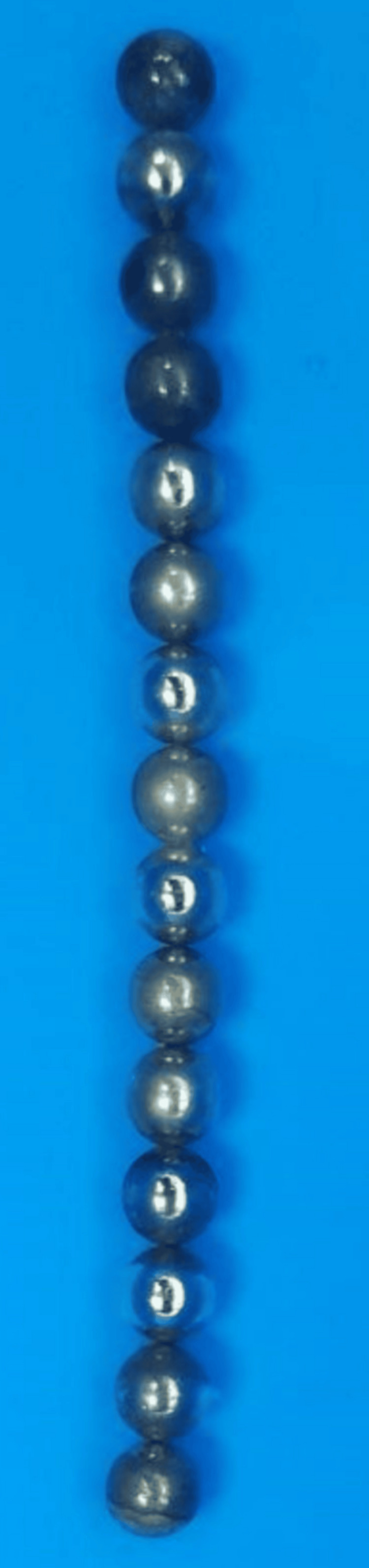
Earth magnets, brought in by the parents upon further questioning.

Postoperatively, the child was doing well, started oral feeding on day 3, and was discharged home in good general condition the next day. On further questioning, the parents mentioned the presence of magnets (Figure 7) at home, with some currently missing, which could have been easily swallowed by the child.

## Discussion

Enteroenteric fistulas can arise from several etiologies, including underlying diseases like inflammatory bowel disease, postoperative complications, prolonged radiation exposure, malignancies, or foreign body ingestion. These factors compromise the integrity of the intestinal wall, leading to tissue breakdown and perforation, which enables fistula formation. Through this breach, an abnormal connection forms between segments of the intestine, establishing the fistula pathway [1].

Initial assessment should be done in the form of a detailed history and thorough clinical examination of the patient. Based on these findings, the appropriate imaging studies and endoscopy can be used to further evaluate the cause of the fistula, as the initial treatment of this condition is based on treating the underlying trigger, as well as any complications that arise [2-4].

As our patient was completely healthy, with no history of inflammatory bowel disease, malignancy, or previous surgeries, the enteroenteric fistula could only be explained by an unwitnessed magnet ingestion, which caused the fistula and then passed in the feces unnoticed.

With this in mind, foreign body ingestion is a prevalent concern in the pediatric population, particularly between the ages of six months and three years [5]. In fact, magnet ingestion has become a matter of concern in the past decade due to the increased prevalence of magnet toys found in homes. The most common symptoms following ingestion are abdominal pain and vomiting; however, the majority of cases are asymptomatic early on, and the symptoms become more apparent depending on the location and duration post-ingestion [4,6].

Typically, patients present in one of two ways. At first, they are symptomatic, with the magnet identified on imaging, such as an abdominal X-ray, or the ingestion is witnessed by a parent or guardian who brings them to the hospital, prompting immediate intervention. In the second scenario, the patient is asymptomatic, but a caregiver reports witnessing the ingestion of magnets. In such cases, an X-ray is performed to assess the number of ingested magnets and guide management. While a single, small, smooth magnetic foreign body often passes without issue, multiple magnets can lead to severe complications, including bowel perforation, obstruction, peritonitis, or even death [5,7-9].

In our case, the magnet was not visible on imaging, and the only clue from the history was a missing magnet.

## Conclusions

This case highlights the serious potential complications of unwitnessed magnet ingestion in pediatric patients, even when no underlying health conditions are present. This signifies the importance of maintaining a high index of suspicion for foreign body ingestion in young children presenting with nonspecific abdominal symptoms, particularly in households with small magnets accessible to children. Prompt assessment, including thorough history-taking, appropriate imaging, and early intervention, is essential in managing and preventing complications associated with foreign body ingestion.
